# Changes in DNA methylation at the aryl hydrocarbon receptor repressor may be a new biomarker for smoking

**DOI:** 10.1186/1868-7083-5-19

**Published:** 2013-10-11

**Authors:** Robert A Philibert, Steven R H Beach, Man-Kit Lei, Gene H Brody

**Affiliations:** 1 Department of Psychiatry, University of Iowa, Rm 2-126 MEB, Iowa City, IA 52242, USA; 2 The University of Georgia, Athens, GA, USA

**Keywords:** Aryl hydrocarbon receptor repressor, Biomarker, DNA methylation, Epigenetics, Lymphocytes, Smoking

## Abstract

**Background:**

Smoking is the largest preventable cause of morbidity and mortality in the United States. In previous work, we demonstrated that altered DNA methylation at the aryl hydrocarbon receptor repressor (AHRR) is correlated with self-reported smoking in 19-year-old African Americans with relatively low levels of smoking. However, one limitation of the prior work is that it was based on self-reported data only. Therefore, the relationship of AHRR methylation to smoking in older subjects and to indicators such as serum cotinine levels remains unknown. To address this question, we examined the relationship between genome- wide DNA methylation and smoking status as indicated by serum cotinine levels in a cohort of 22-year-old African American men.

**Results:**

Consistent with prior findings, smoking was associated with significant DNA demethylation at two distinct loci within AHRR (cg05575921 and cg21161138) with the degree of demethylation being greater than that observed in the prior cohort of 19-year-old smoking subjects. Additionally, methylation status at the AHRR residue interrogated by cg05575921 was highly correlated with serum cotinine levels (adjusted *R*^2^ = 0.42, *P* < 0.0001).

**Conclusions:**

We conclude that AHRR DNA methylation status is a sensitive marker of smoking history and could serve as a biomarker of smoking that could supplement self-report or existing biomarker measures in clinical or epidemiological analyses of the effects of smoking. In addition, if properly configured as a clinical assay, the determination of AHRR methylation could also be used as a screening tool in efforts to target antismoking interventions to nascent smokers in the early phases of smoking.

## Background

Cigarette smoking is a leading preventable cause of mortality in the United States and leads to the premature death of over 100,000 Americans each year [1]. Despite substantial public and private sector efforts to decrease the rate of smoking, the rate of smoking in US adults remains at approximately 19% [2]. To date, efforts to decrease smoking have taken two forms [3]. The first strategy focuses on changes in public policy designed to decrease the availability of cigarettes or to educate the public on the adverse consequences of smoking. The second seeks to increase the effectiveness of smoking cessation treatment. Both of these approaches have had their share of success in decreasing the rate of smoking from 43% in 1965 to current levels [4]. However, despite ongoing efforts, the rate of smoking in young adults has largely stabilized and additional advances are needed to further decrease the rate of smoking.

Conceivably, a better biomarker for smoking could increase the effectiveness of preventive interventions. Smoking prevention programming depends on sensitive and valid epidemiological surveillance of the processes surrounding smoking initiation. Currently, many of these analyses are solely dependent on self-report data, which can be inaccurate. Therefore, it is important that the field develop new tools to supplement existing self-report and existing biomarkers of this critical period.

A better biomarker for smoking could also improve efforts to treat patients in the early phases of smoking. Like most addictive behaviors, smoking is most effectively treated in the first two stages of use, smoking initiation and periodic smoking [5]. In these early stages, smoking cessation efforts may be less hindered by well-established patterns, cues, and symptoms of withdrawal. Unfortunately, identifying individuals in these two earliest stages of smoking, initial experimentation and experimental smoking, is somewhat difficult. Currently, the principal mode of identifying these early stage smokers is through self-reporting. Despite its general utility in a research context, there are concerns about the reliability of self-reported data, particularly if nascent smokers do not wish to be identified or are embarrassed about their smoking [6,7]. Objective measures, namely serum cotinine and carbon monoxide assessment, are effective in identifying individuals who are in the more advanced regular and dependent phases of smoking [8]. However, owing to the restricted detection windows for cotinine and carbon monoxide measurements, these same biomarkers are often insensitive in earlier stage smokers or in the so-called ‘chippers’, smokers who only smoke at weekends [8]. Hence, a more sensitive marker of early onset smoking could conceivably aid efforts to treat early onset smoking by increasing our ability to detect the more malleable, earlier phases of cigarette use.

It is possible that by detecting smoking associated changes in DNA methylation, we may devise a better method to detect the early phases of smoking. Recently, we and others have demonstrated that established smoking is associated with altered DNA methylation at a number of loci, including AHRR, MYO1G, and GFI1 [9-12]. However, these studies greatly differed from each another in the chronicity of smoking and the type of DNA being assessed. Based on our prior study of 19-year-old African American males and self-reported data, we believe that demethylation at the CpG residue in the aryl hydrocarbon receptor repressor (AHRR) recognized by cg05575921, may be the first change evident in the methylome [13]. If so, change at this locus may be an excellent indicator of nascent smoking and further smoking could be expected to both increase the amount of demethylation at this locus and be accompanied by additional changes in the genome. In this communication, we expand on our previous study of 19-year-old male smokers by using a slightly older population (22 years of age) of male subjects and objective measures of smoking detection to re-examine the relationship of smoking to genome-wide methylation.

## Results

The clinical and demographic characteristics of the 107 ‘Adults in the Making’ (AIM) program subjects who participated in the study are given in Table 1. The subjects averaged 22 years of age. Nearly 54% of the subjects reported having smoked at least one cigarette during our clinical interviews. The amount of self-reported smoking tended to be rather light, with the 35 subjects who reported smoking at the last wave of data reporting an average daily consumption of 8 ± 7 cigarettes.

**Table 1:** Clinical and demographic characteristics of the study subjects

***N***		**107**
Age (years)		22.0 ± 1.3
Self-reported smoking status	Never	49
	Waves 1 to 3 only	23
	Wave 4	35
Average cigarette consumption in wave-4 smokers		8 ± 7 per day
Pack year history in wave-4 smokers	≤1 pack year	24
	1 to 2 pack years	5
	>2 pack years	6
Serum cotinine levels (ng/ml)	<1.0	43
	1 <*x* < 2.0	0
	>2.0 ng/ml	64
Average cotinine level in those with cotinine >2 ng/ml		80 ± 58

**Figure 1:**
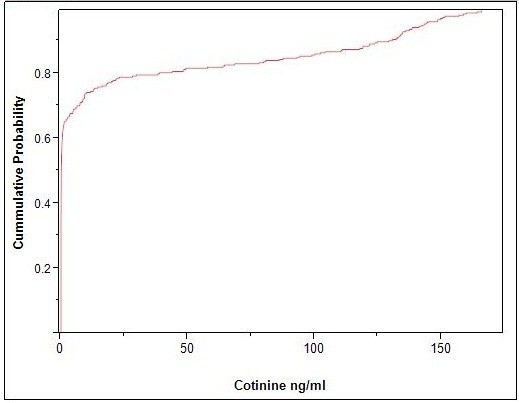
**Cumulative distribution of serum cotinine levels.** The distribution makes a sharp transition above 1 ng/dl with no subjects having values between 1 and 2 ng/dl.

Because our DNA samples were collected approximately 6 months after the collection of wave-4 data and self-reported data may often be an under report of actual smoking consumption [6,7], we next examined serum cotinine levels each of the subjects. Figure 1 illustrates the cumulative frequency distribution of the serum cotinine levels. As the figure illustrates, there was a sharp dogleg break in the distribution of values, with 44 (41%) of the subjects having levels of <1 ng/ml, no subjects having values between 1 and 2 ng/dl and 64 (59%) of the subjects having serum cotinine levels of >2 ng/dl (designated here after as positive cotinine values). Of considerable interest, 23 of the 64 subjects who denied smoking at all four waves including the last interview conducted 6 months prior to the blood draw, had serum cotinine levels of >2.0 ng/dl.

As the first step of our main epigenetic analyses, we conducted genome-wide analysis of the relationship of smoking to DNA methylation. Because the serum cotinine data of Figure 1 suggest that self-reported smoking status may not be reliable, we choose to use serum cotinine levels as our indicator of current smoking status, and contrasted the DNA methylation status of those 64 subjects with serum cotinine levels >2 ng/ml only with that of those 37 subjects who consistently denied smoking through all four waves of data collection and who had negligible levels of serum cotinine (<1.0 ng/ml). Because our previous work with monoamine oxidase A (MAOA) has shown that smoking cessation is associated with a highly variable remodeling of the MAOA DNA methylation signature, the data from the six subjects with serum cotinine levels <1.0 ng/dl but with a positive self-reported history of smoking were not included in the genome-wide contrasts [14].

Table 2 lists the 30 most significant findings with respect to the data from those 98 subjects. Consistent with prior studies, cg05575921 was the probe most highly associated with smoking status with a false discovery rate (FDR) corrected *P* value <0.002 (Nonsmoker (NS) greater than smokers (S); NS mean 0.85, S mean 0.74, 95% confidence interval 0.82 to 0.87, and 0.72 to 0.76, respectively). A second probe from AHRR, cg21161138, also attained genome-wide significance with a FDR corrected *P* value <0.03 (NS greater than S; NS mean 0.73, S mean 0.69, 95% confidence interval 0.72 to 0.75, and 0.68 to 0.70, respectively). Finally, there was a trend for association at a third AHRR probe locus, cg26703534 (NS greater than S; NS mean 0.69, S mean 0.64, 95% confidence interval 0.68 to 0.70, and 0.63 to 0.65, respectively). Methylation at MYO1G probe cg22132788, which Joubert and colleagues [10] had reported to be differentially methylated in DNA prepared from newborns of smoking mothers, was the fourth-ranked probe, with a genome-wide corrected *P* value of <0.144.

**Table 2:** The 30 most significantly associated probes in DNA from men

**Probe ID**	**Gene**	**Placement**	**Island status**	**Average beta values**		***t*** **test**	**Corrected *P *** **value**
				**Smoker**	**Nonsmoker**		
cg05575921	AHRR	Body	N shore	0.74	0.85	4.92 × 10^−9^	0.002
cg21161138	AHRR	Body		0.69	0.73	1.18 × 10^−7^	0.029
cg26703534	AHRR	Body	S shelf	0.64	0.69	4.72 × 10^−7^	0.076
cg22132788	MYO1G	Body	Island	0.94	0.88	1.19 × 10^−6^	0.144
cg17072268	PLD3	TSS1500	N shore	0.82	0.84	1.11 × 10^−5^	0.999
cg12108912	TMEM177	TSS1500	N shore	0.79	0.80	1.33 × 10^−5^	0.999
cg12803068	MYO1G	Body	S shore	0.83	0.76	1.61 × 10^−5^	0.999
cg22904815			N shore	0.44	0.48	1.65 × 10^−5^	0.999
cg25628057	ATAD3B	Body	S shore	0.87	0.88	3.04 × 10^−5^	0.999
cg04521543	TMEM18	3'UTR		0.83	0.82	3.29 × 10^−5^	0.999
cg11270237			N shore	0.34	0.36	3.61 × 10^−5^	0.999
cg00498653			Island	0.15	0.17	3.80 × 10^−5^	0.999
cg22537081	TBRG4	TSS200	Island	0.03	0.03	3.94 × 10^−5^	0.999
cg23311108				0.33	0.36	5.29 × 10^−5^	0.999
cg27312872	C1orf212	Body	N shelf	0.83	0.84	5.50 × 10^−5^	0.999
cg13960339	ZIM2	TSS200	Island	0.51	0.53	6.27 × 10^−5^	0.999
cg07918390	GPSM3	TSS1500	Island	0.04		6.60 × 10^−5^	0.999
cg16148833				0.72	0.74	6.85 × 10^−5^	0.999
cg27072683	NDUFB8	TSS1500	S shore	0.25	0.27	7.28 × 10^−5^	0.999
cg16579844	RNASE4	1stExon	S shore	0.04	0.04	7.54 × 10^−5^	0.999
cg08939942				0.91	0.92	7.59 × 10^−5^	0.999
cg25202390	MRPL30	1stExon	Island	0.18	0.16	9.23 × 10^−5^	0.999
cg04097463			S shelf	0.84	0.86	9.76 × 10^−5^	0.999
cg19192585			Island	0.03	0.03	9.78 × 10^−5^	0.999
cg18075691			N shelf	0.41	0.36	9.87 × 10^−5^	0.999
cg20215007	ZNF467	5'UTR	N shore	0.19	0.21	0.0002	0.999
cg11467141			Island	0.95	0.92	0.0002	0.999
cg00534919	C1orf26	Body		0.10	0.10	0.000113816	0.999
cg08771171	CTNNA1	Body		0.80	0.82	0.0001182	0.999
cg21029030	MIF4GD	TSS1500	Island	0.02	0.02	0.000118582	0.999

All average methylation values are non-log transformed beta values. Island status refers to the position of the probe relative to the island. Classes include: 1) Island, 2) N (north) shore, 3) S (south) shore, 4) N (north shelf), 5) S (south) shelf and 6) blank, denoting that the probe does not map to an island.

**Figure 2:**
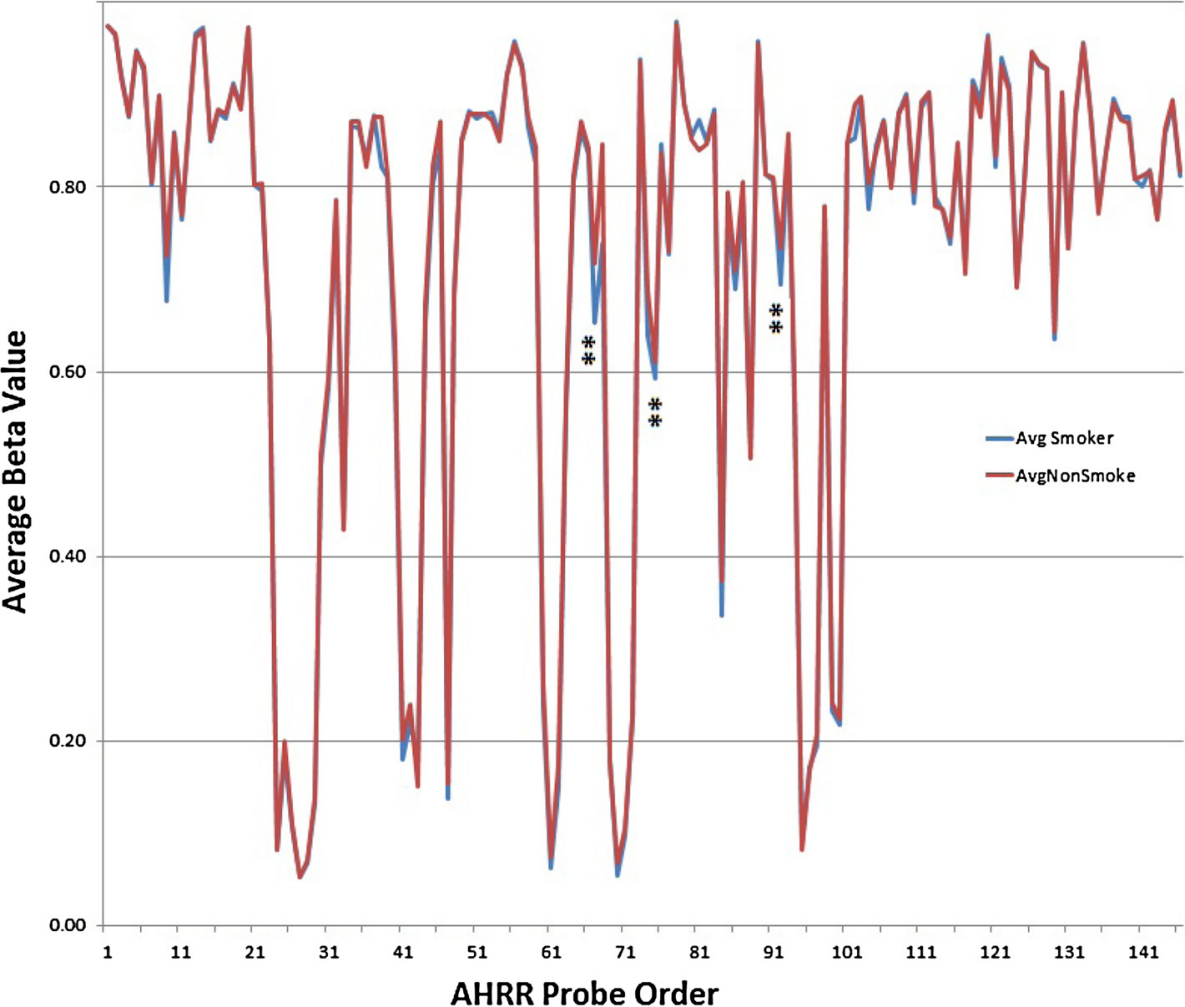
**Comparison of the methylation levels in DNA from male smokers (*n*= 64) and lifetime male nonsmokers (*n*= 37) at the 146 probes covering the AHRR locus.** The average of the nonsmokers is indicated by the red line, whereas the values for smokers when they diverge from that of the nonsmokers as illustrated by blue line. The location of those three AHRR probes with at least a trend for genome-wide significance is illustrated by the double asterisk. The exact ID, methylation values, and *P* values for the comparisons at each probe are given in Additional file 1: Table S1.

Because AHRR is a complexly regulated gene (for example, it has at least five CpG islands) with 146 probes mapping to it, we then scrutinized the relationship of smoking status to methylation at each these 146 probes. Figure 2 illustrates the degree of methylation at each of those residues in the smokers and nonsmokers, while Additional file 1: Table S1 gives the ID, position, sequence exact averages, and *P* values obtained for each probe. As the figure and table together demonstrate, 10 probes clustering to four discrete areas have nominal significance values of < 1×10^-3^. Notably, at all ten of these AHRR probes with a nominal significance value of < 1×10^-3^, smoking was associated with demethylation.

**Figure 3:**
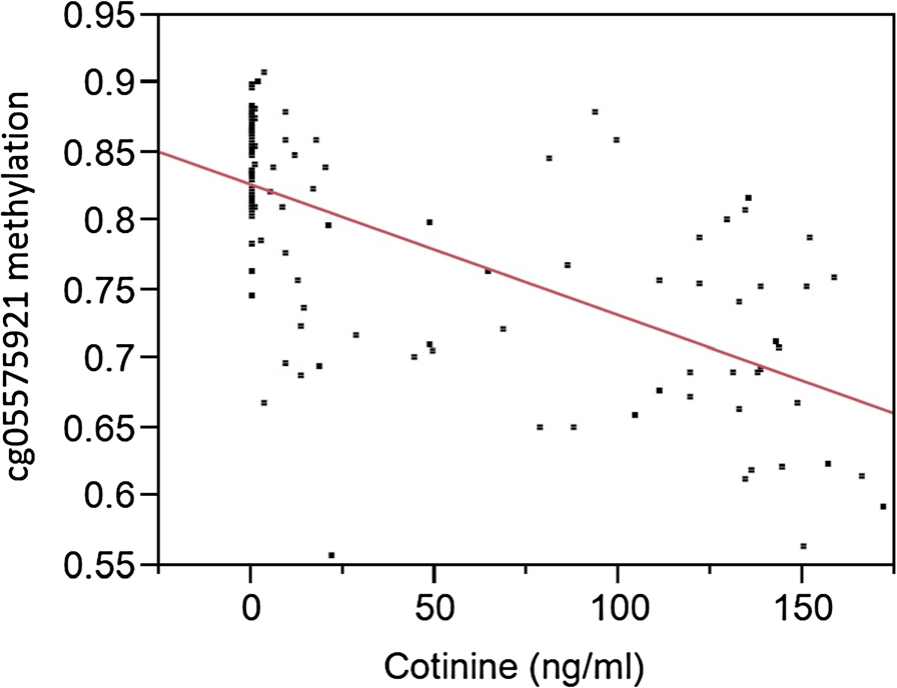
**Relationship between cg05575921 methylation and serum cotinine levels for all 111 subjects.** The methylation of cg05575921 is expressed as the nontransformed beta value, which can be roughly viewed as the percentage of methylation.

Because methylation at cg05575921 was once again the most highly associated residue in terms of DNA methylation, we analyzed the relationship between methylation status at that residue and serum cotinine levels. Using the data from all 107 subjects, we found that methylation status at cg05575921 was highly correlated with serum cotinine levels (Figure 3, adjusted *R*^2^ = 0.42, *P* < 0.0001). Methylation status at the other two highly associated AHRR residues, cg26703534 (adjusted R^2^ = 0.28, *P* < 0.0001) and cg21161138 (adjusted R^2^ = 0.19, *P* < 0.0001), was also highly correlated, although the proportion of the variance explained was considerably less.

## Discussion

Using data from a group African Americans who are slightly older than our previous group of subjects, we confirm and extend our prior findings, showing that AHRR appears to be the locus whose methylation is significantly affected by nascent smoking, with degree of demethylation strongly associated with level of exposure. In addition, we show a strong correlation between demethylation at cg05575921 and serum cotinine levels. Significant limitations of the current study include the reliance on self-reported data for certain aspects of the study and the lack of self-reported data with respect to smoking at the time of the actual blood sampling.

The findings with respect to AHRR extend the prior findings in 19-year-old African American subjects and indicate that smoking induces a steady yet predictable series of changes in the methylation signature of lymphocytes. In our first group of 19-year-old men, only cg05575921 was significantly changed with an average change of 6%. In this group of slightly older subjects, with a presumably longer smoking history, the average demethylation at cg05575921 was 11%, with two other probes from AHRR achieving at least a trend for genome-wide significance. Taken together with other evidence, this suggests that continued smoking increases the degree of change at AHRR and other genes, even though degree of smoking, on average, remained quite low in this slightly older sample. Some other changes may be notable at genes suggested by others, including MYO1G (herein the fourth-ranked probe), F2RL3 and GFI1 [9,10,12]. Indeed, in our analyses of the effects of smoking on DNA methylation in 50-year-old African American smokers, the methylation signatures of a large number of genes are significantly remodeled (Dogan *et al*., unpublished data). Hence, it may be that as individuals continue to smoke, the degree of differential methylation at these other loci continues to develop to the point that it is detectable at genome-wide levels using similarly powered analyses. This also suggests the possibility of dose–response relationships at other CpG sites in addition to those on AHRR.

The semiquantitative nature of the relationship between serum cotinine levels and AHRR methylation status raises the possibility that DNA methylation could be used as a biomarker for smoking in place of exhaled carbon monoxide or serum cotinine levels when such measures are unavailable. Indeed, for large-scale epidemiological work, DNA demethylation at AHRR might prove useful as an index of smoking if there is stored blood or if other potential assessments are unavailable. For those existing data sets without separate serum samples or quantitative smoking data, this is certainly an attractive possibility. In addition, given the relatively short half-life of exhaled carbon dioxide (3 to 5 hours) [15] and serum cotinine levels (15 hours) [8,16], the current data suggest that altered DNA methylation could be used to detect otherwise undetectable smoking by individuals such as ‘chippers’, who smoke only periodically [8,16]. Further research to develop the response profile for AHRR and related loci could result in the development of a versatile assessment tool that could find considerable use in both research and clinical applications.

It is natural to ask why AHRR is the most significant locus. Although not immediately intuitive at first glance, changes in the epigenetic status of AHRR could be expected to be one of the first cellular responses to tobacco smoke exposure, owing to the interaction of AHRR with the aryl hydrocarbon receptor (AHR), which is the induction point for the xenobiotic pathway [17]. This catabolic pathway, which is active both in the liver and in lymphocytes, includes several well-known P450 enzymes, including CYP1A1, and is responsible for the degradation of environment toxins, such as polyaromatic hydrocarbons and dioxins commonly found in cigarettes [18,19]. Activation of the pathway is initiated by the binding of ligands such as dioxin, which also serve as targets for degradation to the PAH domain of AHR. Following ligand binding, the AHR protein dimerizes with the aryl nuclear receptor translocator (ARNT), which facilitates its translocation to the nucleus and to binding to the promoters of key catabolic genes. AHRR serves as a negative feedback regulator of AHR induction and does so by competing with AHR for binding with ARNT and by sterically competing with AHR at critical gene promoters [20]. Critically, changes in AHRR methylation are known to alter AHRR gene expression [11]. Unfortunately, because AHRR has at least 21 known splice variants and 10 known protein isoforms, the relationship between these toxin exposures, AHRR methylation changes, and AHR pathway activity is likely to be complex. However, given the extant data, it is reasonable to hypothesize that the demethylation seen in smokers is associated with increased AHR activation of the xenobiotic pathway, with the current findings highlighting the need for further understanding of these processes.

A pertinent negative in the current study is the failure to observe significant changes in the DNA methylation signature at nicotinic cholinergic receptors (NChRs). However, it is important to note that in contrast to the situation with respect to AHRR, NChRs are not expressed heavily nor are they functionally coupled in lymphocytes. Furthermore, the genome-wide approaches used in this paper are relatively insensitive to smaller scale, yet more behaviorally relevant smoking associated changes in genes, such as monoamine oxidase A (MAOA), which is only lightly expressed in the lymphocytes [14]. Therefore, examinations of the role of smoking associated changes of NChR methylation in addictive processes should perhaps focus on those cell types in which the genes are heavily expressed and functionally coupled.

A potential problem for any epigenetic study is the presence of confounding genetic vulnerability. However, this is not likely to be a problem for our findings with respect to cg05575921, for several reasons. The nearest polymorphisms, rs6869832 and rs6894195, are relatively uninformative in the African American population (minor allele frequency 0.02); in a previous study of 399 subjects, we genotyped these loci and found no effect on cg05575921 methylation [13]. Still, genetic variation may have an effect on the methylation status at other interesting loci and we encourage the reader to inspect Additional file 1: Table S1 carefully for further details on polymorphisms flanking potentially interesting CpG residues.

An unanticipated finding was the degree of disparity between self-reported smoking status at wave 4 and the serum cotinine levels determined using samples collected 6 months after wave-4 self-reported data collection. Some discrepancy is, of course, understandable. Because the reliability of recall dims with increasing time, and because our yearly examinations only interrogated smoking behavior over the past month, some inaccuracy of self-reporting is to be expected. At the same time, such problems are common in both investigations of adolescent, nascent smoking [6,7] and in studies of smoking in minority populations [21], highlighting the need for biochemical confirmation of smoking status in studies of tobacco use. In addition, some of the disparity between negative self-report and positive cotinine levels may reflect recent onset in smoking.

Our choice of a 2 ng/ml cutoff level was based on analyses of the shape of the cumulative distribution curve. This level is quite consistent with the optimum cutoff levels developed by Benowitz and colleagues using data from 16,156 subjects from the National Health and Nutrition Examination Study (NHANES) [22]. However, it is possible that a few of our lower ‘positive’ cotinine levels reflected secondhand smoke exposure in the home or from friends who smoked. However, in our opinion, secondhand smoke exposure is unlikely to explain more than one or two false-positives. The lowest cotinine level in the self-reported nonsmokers who had serum cotinine levels of >1.0 ng/dl was 9.3 ng/dl, which is considerably above that expected for secondhand smoke exposure [23]. Accordingly, the finding that one-third of the subjects with positive cotinine levels denied smoking at wave 4 suggests either a surge of smoking initiation at this age, or the possibility that both substantive intermittent, fast-moving changes in smoking behaviors and resulting unreliable self-reporting account for the discrepancies. Given the later onset of smoking in African Americans [24] and the higher rates of discrepant reports in underserved minorities [6,21], these findings reemphasize the need for repeated measures with shorter lags between assessments and the need for use of biomarkers in both phenomenological and biological examinations of the effects of smoking. In this context, AHRR emerges as a potentially useful adjunct to self-reporting of smoking and may have particular utility in studies of the early phases of smoking.

## Conclusions

In summary, we confirm and extend prior findings indicating the primacy of the AHRR locus in the epigenetic response to cigarette smoking. We also demonstrate a strong correlation between demethylation of discrete AHRR CpG residues and serum cotinine levels. We suggest that studies to firmly delineate the dose dependency and temporal characteristics of AHRR methylation changes with respect to smoking are indicated.

## Availability of supporting data

The complete data for the AHRR locus are attached as Additional file 1: Table S1.

## Methods

The 107 subjects featured in these analyses are drawn from the AIM project which is a longitudinal study of young African Americans as they transition from adolescence into early adulthood [25]. Youths were enrolled in the study when they were 16 years of age. At wave 1, among youths’ families, median household gross monthly income was below $2,100 and mean monthly per capita gross income was below $900. Accordingly, on average, they could be described as working poor.

### Procedures

Families were contacted and enrolled by community liaisons residing in the counties where the participants lived. The community liaisons were African American community members who worked with the researchers on participant recruitment and retention. At all data collection points, parents gave written consent to minor youths’ participation, and youth gave written assent or consent to their own participation. To enhance rapport and cultural understanding, African American university students and community members served as field researchers to collect data. At the home visit, self-report questionnaires were administered privately via audio computer-assisted self-interviewing technology on a laptop computer. Youths were compensated for their participation with $50 after each assessment. All protocols and procedures used in the AIM project were approved by the University of Georgia Institutional Review Board.

As a part of the self-report assessment, at each wave of data collection, the subjects were asked, ‘In the past month, how often did you smoke cigarettes?’ The number of cigarettes given in reply was used as that year’s estimated average monthly consumption with that number being divided by 20 to give the number of packs smoked. A positive response at any time point from a subject resulted in the categorization of that subject as a smoker for the given wave.

Approximately 6 months after the collection of the wave-4 data, the subjects were phlebotomized to provide sera and DNA for the proposed studies. Their average age was 22. The DNA for the current studies was prepared from lymphocyte (mononuclear) cell pellets, as previously described [13]. Sera were prepared using serum separator tubes and were frozen at −80°C after preparation until use.

Genome-wide DNA methylation was assessed using the Illumina (San Diego, CA) HumanMethylation450 Beadchip by the University of Minnesota Genome Center (Minneapolis, MN) using the protocol specified by the manufacturer as previously described [26]. This chip contains 485,577 probes recognizing at least 20216 transcripts, potential transcripts or CpG islands. Subjects were randomly assigned to 12 sample ‘slides’ with groups of eight slides representing the samples from a single 96-well plate being bisulfite converted in a single batch. Four replicates of the same DNA sample were also included to monitor for slide-to-slide and batch bisulfite conversion variability with the average correlation co-efficient between the replicate samples being 0.997. The resulting data were inspected for complete bisulfite conversion and average beta values for each targeted CpG residue determined using the Illumina Genome Studio Methylation Module, Version 3.2. The resulting data were then cleaned using a Perl-based algorithm to remove those beta values whose detection *P* values, an index of the likelihood that the observed sequence represents random noise, were greater than 0.05.

Genome-wide linear regression analyses of the log transformed data were conducted using MethLAB, version 1.5, using our previously described procedures [13,27]. All the analyses were controlled for both batch and slide. Correction for multiple comparisons was accomplished by using the false discovery rate method using an alpha of 0.05 and a subroutine within MethLAB [28]. As noted in the results, the regression analyses that were controlled for batch and slide contrasted the log transformed beta values of those who denied ever having smoked and had serum cotinine levels <1.0 ng/dl (*n* = 37) with those with serum cotinine levels >2.0 ng/dl (*n* = 64).

The analyses of clinical, serological and single point methylation data were analyzed using the suite of general linear model algorithms contained in JMP, version 10 (SAS Institute, Cary, USA), as indicated in the text.

## Abbreviations

AHR: Aryl hydrocarbon receptor; AHRR: Aryl hydrocarbon receptor repressor; AIM: Adults in the making; ARNT: Aryl hydrocarbon nuclear translocator; FDR: False discovery rate; MAOA: Monoamine oxidase A; NChR: Nicotinic cholinergic receptors; NS: Nonsmoker; S: Smoker.

## Competing interests

The University of Iowa filed intellectual property right claims and has transferred some of those rights on some of the material related to this manuscript to Dr. Philibert. Dr. Philibert is also the Chief Scientific Officer and partial owner of Behavioral Diagnostics, which has a funded NIH application with respect to the use of methylation to detect alcohol use (R43AA022041). Drs. Beach and Brody do not have any conflicts to disclose.

## Authors’ contributions

RAP conducted the initial genome-wide analyses and serum cotinine assessments, and wrote the initial draft of the manuscript. M-KL and SRHB assisted in the analyses and in writing the manuscript. GHB conceptualized the framework of the AIM studies, supervised the collection of clinical data, and assisted in writing the manuscript. All authors read and approved the final manuscript.

## Supplementary Material

Additional file 1: Table S1 This file contains the beta values for all 107 subject for every locus in AHRR as well as the annotation file which contains extensive information with respect to probe sequence, relative gene location, local genetic variation, etc. Click here for file
